# Self-Stigma and Its Relationship with Victimization, Psychotic Symptoms and Self-Esteem among People with Schizophrenia Spectrum Disorders

**DOI:** 10.1371/journal.pone.0149763

**Published:** 2016-10-26

**Authors:** Ellen M. A. Horsselenberg, Jooske T. van Busschbach, Andre Aleman, Gerdine H. M. Pijnenborg

**Affiliations:** 1 Department of Psychotic Disorders, GGZ Drenthe, Assen, The Netherlands; 2 University of Groningen, University Medical Center Groningen, Rob Giel Research Center, Groningen The Netherlands; 3 Windesheim University of Applied Sciences, Zwolle, The Netherlands; 4 Department of Neurosciences, University Groningen, University Medical Center Groningen, Groningen, The Netherlands; 5 Department of Clinical Psychology and Experimental Psychopathology, Faculty of Behavioural and Social Sciences, University Groningen, Groningen, The Netherlands; Katholieke Universiteit Leuven, BELGIUM

## Abstract

**Objective:**

Self-stigma is highly prevalent in schizophrenia and can be seen as an important factor leading to low self-esteem. It is however unclear how psychological factors and actual adverse events contribute to self-stigma. This study empirically examines how symptom severity and the experience of being victimized affect both self-stigma and self-esteem.

**Methods:**

Persons with a schizophrenia spectrum disorder (N = 102) were assessed with a battery of self-rating questionnaires and interviews. Structural equation modelling (SEM) was subsequently applied to test the fit of three models: a model with symptoms and victimization as direct predictors of self-stigma and negative self-esteem, a model with an indirect effect for symptoms mediated by victimization and a third model with a direct effect for negative symptoms and an indirect effect for positive symptoms mediated by victimization.

**Results:**

Results showed good model fit for the direct effects of both symptoms and victimization: both lead to an increase of self-stigma and subsequent negative self-esteem. Negative symptoms had a direct association with self-stigma, while the relationship between positive symptoms and self-stigma was mediated by victimization.

**Conclusions:**

Our findings suggest that symptoms and victimization may contribute to self-stigma, leading to negative self-esteem in individuals with a schizophrenia spectrum disorder. Especially for patients with positive symptoms victimization seems to be an important factor in developing self-stigma. Given the burden of self-stigma on patients and the constraining effects on societal participation and service use, interventions targeting victimization as well as self-stigma are needed.

## Introduction

In addition to the burden of having a severe mental illness, people with schizophrenia often encounter stigmatization [1]. Such stigmatization does not only occur through others and society, but patients may also have self-stigmatizing thoughts themselves. Self-stigma (i.e. internalized stigma) arises when people with a mental disorder are aware of negative stereotypes associated with their diagnosis, internalize these stereotypes and apply them to themselves [2]. Almost half of the people (41.7%) with schizophrenia or other psychotic disorders report to experience self-stigma [3]. Self-stigma may undermine the person’s sense of self-esteem and exerts influence on peoples’ ability to pursue behaviours related to important life goals, which is also referred to as the ‘Why Try’ effect [4]. The ‘why try’ effect elaborates on modified labelling theory by outlining how low self-esteem and self-efficacy also affects service participation. In turn low service participation in evidence-based practices can lead to more symptoms and less goal attainment. Recent research also suggests that in young people at risk for psychosis self-stigma can influence the transition to schizophrenia after one year [5].

Since not everyone with a psychotic disorder will develop self-stigma it is important to understand what factors, i.e. social, psychological and psychiatric, contribute and predict people experiencing more or less self-stigma. A recent meta-analysis including studies with both cross-sectional and longitudinal research designs shows a consistent relationship of self-stigma with psychosocial variables, including hope, self-esteem and empowerment, having significant and negative associations with internalized stigma. Regarding psychiatric variables, symptom severity was positively associated with internalized stigma. Longitudinal studies offered preliminary evidence suggesting that positive symptoms serve as a predictor of internalized stigma over time [6]. Lysaker et al. [7] reported self-stigma to be associated with higher levels of positive symptoms, but no association with negative symptoms, disconfirming earlier research linking negative symptoms with discrimination [8]. However, other studies did find a strong positive association between internalized stigma and negative symptoms [9]. Research by Cavelti et al. [10], building on the work of Yanos et al [11] who tried to unravel the direction of the association between symptoms and self-stigma, showed that causality between symptom severity and self-stigma is complex and remains unclear.

Most research on the precursors of self-stigma has focused on psychological factors. However, the influence of (adverse) environmental factors, such as victimization, has been underexposed. One study incorporated external/contextual factors (i.e. location, activity, social company) showing that there is substantial variation in the experience of self-stigma within persons, and these fluctuations are related to current activities, mood and symptoms [12]. Increases in doing unspecified ‘other’ activities, negative affect and psychotic symptom severity predicted increases in self-stigma. So self-stigma seems an experience that changes based on alterations in internal states and external circumstances. In this respect the influence of victimization has not been studied, until now. Victimization includes overt violence causing physical harm, but also aggressive social behaviour like rude jokes and scolding. The prevalence of victimization for individuals with severe mental disorders like schizophrenia is ten times higher than in the general population, even after controlling for demographic characteristics [13–18]. 81% of individuals with schizophrenia are exposed to at least one traumatic event, with an average of 3.5 different types of traumatic events experienced during the lifetime [15]. Rates of victimization across different studies range from 8% till 34% [19] and vary considerably as a consequence of varying incident periods and heterogeneous patient groups.

Research in the area of victimization and severe mental illness is limited and complex with a lot of mixed results. There are clear indications of a relationship between victimization and course and outcome [20, 21]. But, where victimization is consistently associated with severity of positive symptoms [16, 18, 22–25] empirical studies show mixed results for the association with negative symptoms [18, 19]. Furthermore manic symptoms seem to increase the risk of victimization in patients with schizophrenia [26]. However, no research is available yet expanding the ‘why try’ effect by including environmental factors like victimization as precursors of self-stigma. It can be hypothesized that people who are victimized are at greater risk of developing self-stigma. There is considerable evidence that victims in general experience a marked decrease in their sense of self-worth [27]. They perceive themselves as having been singled out for misfortune leading to self-questioning, a perception of deviance and self-stigma [28]. The reverse direction is also possible: people with self-stigma are less able to assert or defend themselves in interactions with others, thus reinforcing being victimized [29], or they may lack a social network that could deter victimization. As for the interplay between symptom level, victimization and self-stigma, one could argue that people who have more symptoms overall are at higher risk for both being victimized and developing self-stigma. However, there is the possibility of a difference between people with negative symptoms who withdraw from their environment experiencing less victimization than people with positive symptoms. The latter group more often displays strange behavior and may be more focused on their internal states with fewer cognitive resources available to be devoted to interactions with other people, which makes them more vulnerable for victimization.

The aim of our study is to examine this possible interplay between victimization, symptoms, self-stigma and self-esteem. We hypothesized that symptom severity and the experience of being victimized affects an individual’s internalized stigma (self-stigma) and self-esteem. However, whereas we expect a direct effect of negative symptoms on self-stigma, the influence of positive symptoms on self-stigma was hypothesized to be also mediated through victimization.

## Methods

### Participants, recruitment and procedure

102 people (77 men and 25 women) with diagnoses of schizophrenia spectrum disorders (95 with schizophrenia, 5 with schizoaffective disorder, 1 with schizophreniform disorder and 1 with psychotic disorder NOS), confirmed with the Structured Clinical Interview for DSM IV [30] participated in our study. They were recruited from two previous studies from our group: the REFLEX study [31], a multicenter Randomized Controlled Trial studying a social-cognitive group treatment to improve insight in schizophrenia (N = 89), and the EMOZIE study [32]: Insight in affective versus non-affective psychosis: an fMRI study (N = 17). Inclusion criteria consisted of having a diagnosis of a schizophrenia spectrum disorder according to DSM IV-criteria, > eighteen years old and being able to give informed consent. The presence of an acute psychotic episode, a co-morbid neurological disorder and no competence of the Dutch language were reasons for exclusion. The study was approved of by the Medical Ethical Board of the University Medical Centre as an amendment of the Reflex study (METc 2009.220) and the EMOZIE study (METc 2008.305). After complete description of the study and procedures to the participants, written informed consent was obtained. Participants had a mean ± SD age of 39.1±11.3 and a mean education of 15.6±3.41 years. Their illness duration is 13.3±10.3 years and the psychotic episodes 5.03±11.3. All received outpatient treatment, 92 (90%) patients used antipsychotic medication, 10 did not (10%). Data on employed was available for 99 (97%) of participants, 64 (63%) was unemployed, 35 (34%) had payed employment of some sort but only eight participants were in fulltime jobs. The study was conducted between April 2011 until March 2012.

### Measures

#### Self-stigma

Self-stigma was measured by the Internalized Stigma of Mental Illness Scale–[33]. It is a 29-item questionnaire designed to assess subjective experiences of self-stigma using a total score and five subscale scores; alienation, stereotype endorsement, perceived discrimination, social withdrawal and stigma resistance. Participants answer on a four-point agreement scale (4 = strongly agree). Recent research suggests that ‘stigma resistance’ is a separate construct [34] so this subscale was not included in the summed average of the other four ISMI-subscales. Psychometric properties of the ISMIS are good with acceptable internal consistency (α = .90) and test-retest reliability (r = .92) [35].

#### Symptoms

The Positive and Negative Syndrome Scale (PANSS) [36] is a 30-item semi-structured interview completed by clinically trained staff. It was used to assess psychopathological symptoms common in schizophrenia and it consists of three subscales (positive symptoms, negative symptoms and global psychopathology symptoms). For our analyses we only used the positive and negative subscale.

#### Victimization

To assess the prevalence of victimization in the last three years four questions originated from the national crime victimization scale of the ‘Integrale Veiligheidsmonitor’ or IVM (Integral Safety/Security Monitor) [37, 38], the Dutch equivalent of the International Crime Victimization Survey [39], were used referring to being verbally or physically threatened with violence and to being a victim of theft or vandalism and sexual harassment or assault. Participants have to describe the incident and report detailed information on the time, location and perpetrator. There are no data on the reliability and validity of the IVM in the general population, Analysis on data from a cohort of 581 people with psychosis showed no significant relation (corr.093) between paranoid thinking and the reported incidence of victimization for violent events, threats and property crime [40].

#### Self-esteem

Self-esteem was assessed with the Self-Esteem Rating Scale-Short Form (SERS-SF) [41]. The SERS is a 20-item self-rating scale with two subscales: positive and negative self-esteem. The SERS includes statements that are linked to social contacts, as well as achievements and competency. Items are rated on a 7-point “never” to “always” scale. For our analysis we used the subscales separately. The scale has good internal consistency, good test-retest reliability and convergent validity in patients with schizophrenia [41].

## Statistical Analyses

### Three models

Using Structural Equation Modelling (SEM) three different models were fitted on the data (SI Datafile selfstigma and victimization) to test our hypotheses on the mediating effect of victimization on self-stigma especially in the case of positive symptoms and the subsequent effect of self-stigma on self-esteem. The test of the ‘original’ model (1) with direct effects for both symptoms and victimization on self-stigma and a direct effect of self-stigma on self-esteem was used as a reference. This model represents the state of the literature in the most general way. Thereupon a second slightly altered model was tested based on the assumption that not the symptom level itself influences self-stigma but adverse events like experiences with violence and criminal acts. The fit of this model with victimization mediating both positive and negative symptoms (2) was compared to the original. In a third step (model 3) different mechanisms for negative and positive symptoms were specified. For negative symptoms a direct influence on self-stigma was assumed with a lesser chance of victimisation as a mediator because of withdrawal and fewer social interactions. With positive symptoms no direct effect on self-stigma was expected because of lack of insight but self-stigma was supposed to be affected by an increased prevalence of incidents. The stepwise approach was chosen to gain insight in the additional value of the specific hypotheses.

#### Steps before SEM

Several descriptive statistics and correlational analyses using SPSS for Windows, Release Version 19.0, were done before the SEM to examine the association between variables to be included in the model, minimize the number of variables in the analysis and avoid multicollinearity. Since SEM analyses permits no missing data, when necessary (sub) scale totals were generated imputing mean scores for item scores with the restricting of no more than two items missing per (sub) scale.

#### SEM

SEM involves developing a theoretical model to specify causal relationships between constructs (i.e., latent variables) and testing these hypotheses by exploring how well the theoretical model explains the pattern of inter correlations found among the observed variables (i.e., indicator variables). It uses a combination of confirmatory factor analysis and multiple regression in which the whole hypothesized model can be statistically tested in a simultaneous analysis of all the constructs and measured variables [42].

The extent to which the three theoretical models fitted the data was quantified using the χ2 test [43]. A non-significant p-value (p>0.05) and the ratio of χ2/df< 2 would represent an adequate model fit. However, because the χ2 is very sensitive to sample size, it often rejects good-fitting models [42]. Therefore, to provide for reliable evaluations of the model, the Comparative Fit Index (CFI>0.95) [43], Tucker-Lewis Index (TLI>0.95) [44], Root Mean Square Error of Approximation (RMSEA< 0.05) [45], and Standardised Root Mean Square Residual (SRMR<0.05) [44] were also included. To compare the competing models the Akaike Information Criterion (AIC) was used, with smaller values indicating a better model fit [42]. All SEM statistics were conducted with the software package Mplus 5.1.

## Results

Correlation coefficients and the descriptive outcomes of the indicator variables included in the SEM are presented in Table 1. Nearly half of the participants (43.1%) had been victimized in the last three years with robbery (26.5%) mentioned most frequently. Measures on self-stigma were below the midpoint pointing to rather low levels of self-stigma. Symptom levels were almost the same for positive and negative symptoms and both were rather low, typical for individuals not in an active phase of psychosis. Positive as well as negative self-esteem levels were moderate. All correlations were highly significant, except for the correlations between symptoms and negative self-esteem.

**Table 1:** Correlation coefficients and descriptive statistics of variables included in the SEM (n = 102).

Pearson correlations	1	2	3	4	5	M (SD)/%	Ranges
1. PANSS—positive	1					15.31 (4.77)	7–49
2. PANSS-negative	.26**	1				14.17 (5.41)	7–49
3. VICT-total	.31**	.24*	1	1		43.1%	0–1
4 ISMIS-total	.20*	.32**	.29**	-.53**	1	1.65 (0.39)	1–4
5. SERS-SF-Positive	-.24*	-.26**	-.20*	64**	-.46**	46.83 (9.82)	10–70
6. SERS-SF-Negative	.16	.19	.25*			31.87 (11.37)	10–70

* p<0.05,

** p<0.01

**Fig 1:**
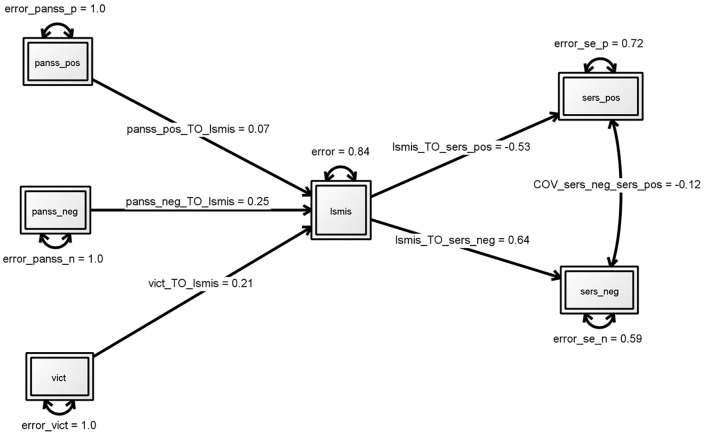
First model: Direct effects symptoms and victimization on stigma and effect of self- stigma on self-esteem. Structural equation modeling. Numbers by single-headed arrows reflect standardized regression weights. Fit indices: χ2 = 3.953; df = 6; p = .6830, RMSEA (90%CI) = .000 (0-.1), SRMR = .036, CFI = 1.000, TLI = 1.041, Akaike (AIC) 3602.784. * p<0.05, ** p<0.01. PANSS_pos: level of positive symptoms on PANSS, PANSS_neg: level of negative symptoms. ISMIS: total score on four subscales of the Internalized Stigma of Mental Illness Scale (alienation, stereotype endorsement, perceived discrimination, social withdrawal) indicating level of self-stigma. SERS_pos: score on subscale positive self-esteem of the Self-Esteem Rating Scale-Short Form and SERS-neg: subscale negative self-esteem.

Results of the SEM for testing our hypothesized first model are presented in Fig 1 with Table 2 showing the (standardized) effects of the different parameters. The fit indices indicate that this original model with direct effects for both symptoms and victimization on stigma and a subsequent effect of self- stigma on self-esteem fits the data already well. Negative symptom levels and being victimized in the last three years significantly relates to self-stigma which in turn has a significantly deteriorating effect on both positive and negative self-esteem. However, no significant effect was found for the influence of positive symptoms on self-stigma (β = .07, p = 0.49).

**Table 2:** (Standardized) effects in in the original hypothesized model (Model 1) and in the new model (Model 3).

	Model 1			Model 3		
	B (SE)	β		B (SE)	β	
Pos sympt -> Self- stigma	.16 (.22)	.07				
Neg sympt -> Self- stigma	.53 (.20)	.25	**	.55 (.19)	.30	**
Victimization -> Self- stigma	4.77 (2.17)	.21	*	5.15 (2.10)	.27	**
Pos sympt -> Victimization				.03 (.01)	.23	*
Self-stigma -> Pos. self-esteem	-.46 (.07)	-.53	**	-.46 (.07)	-.52	**
Self-stigma -> Neg. self-esteem	.65 (.08)	.64	**	.65 (.08)	.63	**
Neg self-esteem <-> Pos self-esteem	-13.15 (7.25)	-.18		-13.15 (7.25)	-.18	

* p<0.05,

** p<0.01

**Fig 2:**
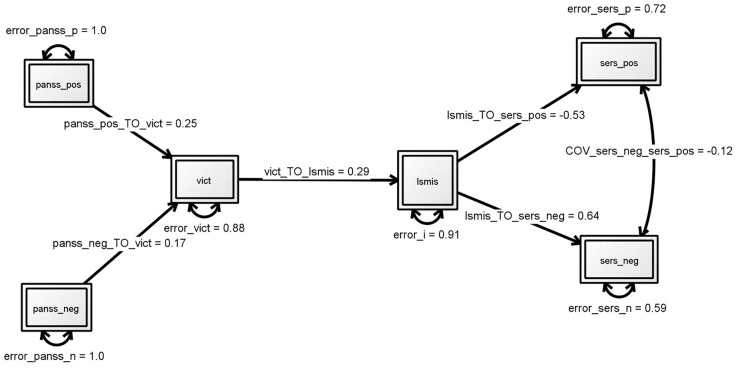
Second model: victimization mediating effects of symptoms on self-stigma and effect self- stigma on self-esteem. Structural equation modeling. Numbers by single-headed arrows reflect standardized regression weights. Fit indices: χ2 = 12.272; df = 8; p = .1395, RMSEA (90%CI) = .072 (0-.128), SRMR = .0086, CFI = .961, TLI = .932, Akaike (AIC) 3615.103. * p<0.05, ** p<0.01. PANSS_pos: level of positive symptoms on PANSS, PANSS_neg: level of negative symptoms. ISMIS: total score on four subscales of the Internalized Stigma of Mental Illness Scale (alienation, stereotype endorsement, perceived discrimination, social withdrawal) indicating level of self-stigma. SERS_pos:score on subscale positive self-esteem of the Self-Esteem Rating Scale-Short Form and SERS-neg: score on the subscale negative self-esteem.

**Fig 3:**
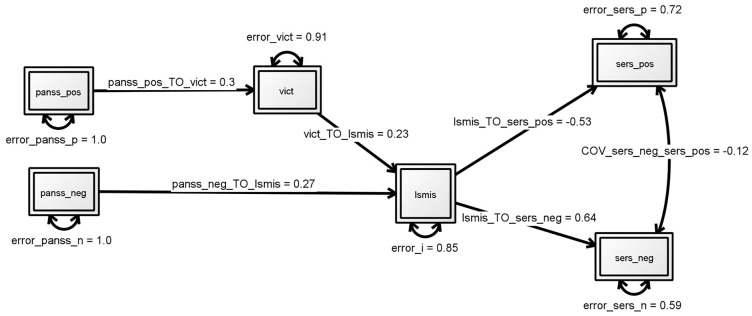
Third model: victimization mediating effects positive symptoms on self-stigma, direct effect negative symptoms on self-stigma. Structural equation modeling. Numbers by single-headed arrows reflect standardized regression weights. Fit indices: χ2 = 7.674; df = 8; p = .4659, RMSEA (90%CI) = .000 (0-.113), SRMR = .0059, CFI = 1.000, TLI = 1.005, Akaike (AIC) 3610.505. * p<0.05, ** p<0.01. PANSS_pos:level of positive symptoms on PANSS, PANSS_neg: evel of negative symptoms. ISMIS: total score on four subscales of the Internalized Stigma of Mental Illness Scale (alienation, stereotype endorsement, perceived discrimination, social withdrawal) indicating level of self-stigma. SERS_pos:score on subscale positive self-esteem of the Self-Esteem Rating Scale-Short Form and SERS-neg:score on the subscale negative self-esteem.

The second model testing the hypothesis that victimization mediates the association of symptoms and self-stigma had no good fit indices: χ2 = 12.272; *df* = 8; *p* = 0.140; RMSEA, 0.072; CFI, 0.961; TLI 0.932, AIC 3615.103 (Fig 2). Parameters showed a significant relation between victimization and positive symptom (β = .0.25, p<0.01) but no such relation for negative symptoms (β = .0.18, p = 0.07). The third model with a direct effect for negative symptoms on self-stigma and an indirect effect for positive symptoms mediated by victimization, again fitted the data well (see Fig 3 and Table 2). Where negative symptoms are directly related to self-stigma (β = .0.27, p<0.01), for positive symptoms the effect is mediated by victimization (β = .29, p<0.01). However, based on a comparison of the goodness of fit indices, this modification showed not to be an improvement over the original hypothesized model (AIC, from 3602.784 to 3610.505).

## Discussion

The present study examined, for the first time, the nature of the relationships between symptoms, victimization, self-stigma and self-esteem among individuals with a schizophrenia spectrum disorder with extra attention to the possible mediating role of especially victimization. Results suggest symptom severity and victimization to be direct predictors of self-stigma. Self-stigma may in turn lead to negative self-esteem. In other words, individuals who reported more severe symptoms and more victimization experiences were found to experience higher self-stigma and lower self-esteem. The current finding of a relationship between symptom severity and self-stigma is consistent with earlier research [6, 7, 9] supporting the hypothesis that more psychotic symptoms may attract attention and be misunderstood as signs of danger or incompetence, thereby making one the target of stigmatization [7,11]. Symptoms like cognitive and social deficits, misperception of social cues and a lack of assertiveness and resilience, partly due to the clinical syndrome, may also make people vulnerable for self-stigma.

However, the direct effect of victimization on self-stigma also draws attention to external factors: not only do people feel that they are different because of their clinical status, the fact that they are confronted with loss of material goods, conflict and violence more often, may also instigate and maintain these feelings. Victimization in this way contributes to a vulnerability of internalizing stigmatizing attitudes.

In our original model a higher level of negative symptoms directly affected self-stigma, but no such direct effect was found for higher levels of positive symptoms. The adapted model showed that positive symptoms do influence self-stigma only through more frequent occurrence of violent incidents. This indicates that people with positive symptoms only develop self-stigma, when they also have been victimized, whereas people with negative symptoms develop self-stigma regardless of being victimized. In this light it is important to note that specifically the first group stands a higher risk of being victimized: several studies found a strong relation with for instance disorganisation [46] and overall severity of psychopathology [47, 48, 49]. Patients with relatively more negative symptoms are likely to withdraw from their environment when experiencing problems with social encounters, avoiding possible victimization, but maybe facilitating internalizing negative stereotypes. On the other hand it has been shown that attitudinal beliefs (that may be closely related to self-stigma) in turn may affect negative symptoms [50]. However the original model still had a better fit than the adapted model including both a direct effect for negative symptoms and an indirect effect for positive symptoms. Notably, however, this partly mediation model was not an improvement of the original model and the fact that both models fitted the data well may indicate that symptoms, victimization and self-stigma exert complex influences over another over time that may not be captured by cross-sectional assessment [7].

With replication, these findings may have clinical implications. The results suggest that any intervention aimed at reducing self-stigma must be placed in a broader context; besides psychological factors, environmental factors such as actual adverse events should also be taken into account. For clinicians, this underscores the importance of screening and monitoring victimization experiences [13] and self-stigma in their patients. To reduce victimization and self-stigma (prevention) programs must be developed and implemented. Regarding the development of interventions to decrease self-stigma, there are broadly two evidence-based interventions; interventions including cognitive behavioural strategies that attempt to alter stigmatizing beliefs and attitudes and interventions encouraging accepting the existence of stigmatizing stereotypes without challenging them, focussing on enhancing stigma coping skills with self-stigma through improvements in self-esteem, empowerment, and help-seeking behaviour [51]. Apart from the preventive influence of adequate symptom management and treatment adherence [52] there are a few interventions specifically aimed at lowering the risk of victimization: psycho-education to risk groups [53] and group interventions focusing on increasing patient safety through discussion with service users [54] or skill-based training with peers [55, 56]. Although many assume that these kinds of interventions should contribute to more knowledge (‘street smart’) and competence ('street skills' to deal with difficult potentially dangerous situations) there is as yet not enough evidence for the effectiveness of these interventions. Thus, evidence-based interventions targeting victimization are needed, making people more resilient and also reducing the likelihood of revictimization, as victimization increases the likelihood of revictimization [57]. Our findings also raise a challenge for integrating strategies targeting both victimization and self-stigma.

Several limitations of our study should be noted. As we used cross-sectional data, we cannot draw definitive conclusions about causality and alternative explanations of the findings cannot be ruled out. For instance, our data did not permit inclusion of the (reverse) effect of violent incidents on symptom level in the model. Longitudinal or prospective studies have better potential to shed a light on these complex interactions and the way time and person characteristics influence their course. This is also important since our sample largely consisted of people with a long history of psychosis and the patterns we found may not be applicable for people with a more recent onset.

Although our sample resembles other samples in this field in terms of age, duration of illness and employment levels [6], mean self-stigma levels were quite low. This could be due to our omission of the stigma resilience subscale. Possibly our recruitment strategy for part of the sample could also have led to an oversampling of people with impaired insight who are known to report less self-stigma since they feel prejudicial statements about psychiatric patients do not apply to them. Future research however should focus on people with high insight when replicating these results, as they probably constitute a high-risk group regarding self-stigma and victimization. In our models the influence of insight was left out all together for methodological reasons. We expected strong multicollinearity between symptoms and insight on the one hand [8] and insight and self-stigma on the other hand [9]. The small sample size also restricted the number of variables to be included in the analysis. However, from other studies we also know that developing self-stigma is mediated by insight which in turn is negatively related to symptom level [10] and some of the explanations offered above can be further elaborated with the influence of insight as an extra explanatory factor. Possibly people with more positive symptoms are more protected against self- stigma since, lacking insight, there is no need to generalize negative stereotypes in society towards themselves until fiercely confronted with serious adverse events. And for people with good insight into the severity of their problems in social communication, affective experience and responsiveness restricting their interaction with others, no violent incidents are necessary to internalize negative stereotypes. For people with lower levels of symptoms, awareness, be it accurate or not, of the difference in opportunities between oneself and those without a diagnosis of schizophrenia can have a devastating effect on self-worth and possibly enhance self-stigma. This contrast can especially been harsh when insight is good and the contrast in day to day life is relatively small.

Furthermore, we were not able to distinguish between different forms of victimization (e.g. verbal, physical, sexual violence) because of the size of the sample and the relatively low prevalence of each of these incidents separately. However, these types of victimization may be differentially related to symptom levels and developing self-stigma. In addition, since participants were predominantly men (75%), associations are may be different in women. Methodological limitations concern the use of self-report measures. Not only can reports of incidents be distorted by cognitive problems, answers can also be biased by earlier experiences on how others reacted on these kind of reports.

## Conclusions

In conclusion, the findings of this study can help to develop a more differentiated view on how not only symptom severity but also adverse events influence the process of self-stigmatisation which in turn has a negative impact on feelings of self-worth and competence for individuals with schizophrenia spectrum disorders. Interventions focusing on symptom reduction, prevention of victimization and empowerment have the potential to reduce self-stigma in patients with schizophrenia spectrum disorder and improving self-esteem. Given the burden of self-stigma on patients and the constraining effects on societal participation and service use, interventions targeting victimization as well as self-stigma are needed and future studies should address the effectiveness of these kinds of interventions.

## Supporting Information

S1 Datafile SI_Datafile selfstigma and victimization. (RTF)

## References

[1] Schulze B, Angermeyer MC: Subjective experiences of stigma. A focus group studyof schizophrenic patients, their relatives and mental health professionals. Social Science & Medicine 56: 299–312, 2003. 12473315 10.1016/s0277-9536(02)00028-x

[2] Corrigan PW, Watson AC, Barr L. The self-stigma of mental illness: implications for self-esteem and self-efficacy. Journal of Social and Clinical Psychology 25: 875–884, 2006.

[3] Brohan E, Rodney E, Sartorius N, Thornicroft G. And for the GAMIAN-Europe Study Group. Self-stigma, empowerment and perceived discrimination among people with schizophrenia in 14 European countries: The GAMIAN-Europe study. Schizophrenia Research 122: 2332–238, 2010. 10.1016/j.schres.2010.02.1065 20347271

[4] Corrigan PW, Larson JE, Rüsch N. Self-stigma and the “why try” effect: impact on life goals and evidence based practices. World Psychiatry 8: 75–81, 2009 19516923 10.1002/j.2051-5545.2009.tb00218.x PMC2694098

[5] Rüsch N, Heekeren K, Theodoridou A, Müller M, Corrigan PW, Mayer B, et al Stigma as a stressor and transition to schizophrenia after one year among young people at risk for psychosis. Schizophrenia Research 166: 43–48, 2015 10.1016/j.schres.2015.05.027 26036814

[6] Livingston JD, Boyd JE. Correlates and consequences of internalized stigma for people living with mental illness: A systematic review and meta-analysis. Social Science & medicine 71: 2150–2161, 2010. 21051128 10.1016/j.socscimed.2010.09.030

[7] Lysaker PH, Davis LW, Warman DM, Strasburger A, Beattie N.. Stigma, social function and symptoms in schizophrenia and schizoaffective disorder: Associations across 6 months. Psychiatry research 149: 89–95, 2007 10.1016/j.psychres.2006.03.007 17156853

[8] Penn DL, Kohlmaier JR, Corrigan PH. Interpersonal factors contributing to the stigma of schizophrenia: social skills perceived attractiveness and symptoms. Schizophrenia Research 45: 37–45, 2000 10978871 10.1016/s0920-9964(99)00213-3

[9] Hill K, Startup M. The relationship between internalized stigma, negative symptoms and social functioning in schizophrenia: The mediating role of self-efficacy. Psychiatry research 206: 151–157, 2013 10.1016/j.psychres.2012.09.056 23218915

[10] Cavelti M, Kvrgic S, Beck EM, Rüsch N, Vauth R. Self-stigma and its relationship with insight, demoralization, and clinical outcome among people with schizophrenia spectrum disorders. Comprehensive Psychiatry 53: 468–479, 2012 10.1016/j.comppsych.2011.08.001 21956043

[11] Yanos PT, Roe D, Markus K, Lysaker PH. Pathways between internalized stigma and outcomes related to recovery in schizophrenia spectrum disorders. Psychiatric Services 59: 1437–1442, 2008 10.1176/appi.ps.59.12.1437 19033171 PMC2605316

[12] Ben-Zeev D, Frounfelker R, Morris BM, Corrigan PW. Predictors of Self-Stigma in Schizophrenia: New Insights Using Mobile Technologies. Journal of dual diagnosis 8(4): 305–314, 2012 10.1080/15504263.2012.723311 23459025 PMC3584451

[13] Teplin LA, McClelland GM, Abram KM, Dulcan MK, Mericle AA. Crime victimization in adults with severe mental illness: comparison with the national crime victimization survey. Archives of General Psychiatry 62: 911–921, 2005 10.1001/archpsyc.62.8.911 16061769 PMC1389236

[14] Choe JY, Teplin LA, Abram KM. Perpetration of violence, violent victimization and severe mental illness: Balancing public health concerns. Psychiatric Services 59: 153–164, 2008 10.1176/appi.ps.59.2.153 18245157 PMC3756896

[15] Mueser KT, Goodman LB, Trumbetta Sl, Rosenberg SD, Osher FC, Vidaver R, et al Trauma and posttraumatic stress disorder in severe mental illness. Journal of Consulting and Clinical Psychology 66: 493–499, 1998 9642887 10.1037//0022-006x.66.3.493

[16] Hiday VA, Swartz M, Swanson JW, Borum R, Wagner HR. Criminal victimization of persons with severe mental illness. Psychiatric Services 50: 62–68, 1999 10.1176/ps.50.1.62 9890581

[17] Maniglio R. Severe mental illness and criminal victimization: systematic review. Acta Psychiatrica Scandinavica 119: 180–191, 2009 10.1111/j.1600-0447.2008.01300.x 19016668

[18] Walsh E, Moran P, Scott C, Burns T, Creed F, Tyrer P, et al Prevalence of violent victimisation in severe mental illness. British Journal of Psychiatry 183: 233–238, 2003 12948997 10.1192/bjp.183.3.233

[19] Fitzgerald PB, de Castella AR, Filia KM, Filia SL, Benitez J, Kulkarni Jl. Victimization of patients with schizophrenia and related disorders. Australian and New Zealand Journal of psychiatry 39: 169–174, 2005 10.1111/j.1440-1614.2005.01539.x 15701066

[20] Brekke JS, Prindle C, Bae SW, Long JD.Risks for individuals with schizophrenia who are living in the community. Psychiatric Services 52: 1358–1366, 2001 10.1176/appi.ps.52.10.1358 11585953

[21] Hiday VA, Swartz M, Swanson JW, Borum R, Wagner HR. Impact of outpatient commitment on victimization of people with severe mental illness. American journal of Psychiatry 159: 1403–1411, 2002 10.1176/appi.ajp.159.8.1403 12153835

[22] Bebbington P, Bhugra T, Singleton N, Singleton N, Farrell M, Jenkins R, et al Psychosis, victimization and childhood disadvantage: evidence from the second British national survey of psychiatric morbidity. British Journal of Psychiatry 185: 220–226, 2004 10.1192/bjp.185.3.220 15339826

[23] Butler R, Mueser K, Sprock J, Braff DL. Positive symptoms of psychosis in posttraumatic stress disorder. Biological Psychology 39: 839–844, 1996. 10.1016/0006-3223(95)00314-2 9172704

[24] Neria Y, Bromet E, Sievers S, Lavelle J, Fochtmann LJ. Trauma exposure and PTSD in psychosis. Journal of consulting and Clinical Psychology 70: 246–251, 2002 11860051 10.1037//0022-006x.70.1.246

[25] Shevlin M, Dorahy MJ, Adamson G. Trauma and psychosis: An analysis of the National Comorbidity Survey. American Journal of Psychiatry 164: 166–169, 2007 10.1176/ajp.2007.164.1.166 17202562

[26] Fortugno F, Katsakou C, Bremner S, Kiejna A, Kjellin L, Nawka P, et al Symptoms associated with victimization in patients with schizophrenia and related disorders. PLoS ONE 8(3): e58142 10.1371/journal.pone.0058142, 2013 23526968 PMC3602443

[27] Krupnick J, Horowitz M. Victims of violence: Psychological responses, treatment implications. Evaluation and change [Special issue: Services for survivors] 42–46, 1980.

[28] Coates D, Winston T. Counteracting the deviance of depression: Peer support groups for victims. Journal of Social Issues 39: 169–194, 1983.

[29] Hodges E, Malone MJ, Perry DG. Individual risk and social risk as interacting determinants of victimization in the peer group. Developmental Psychology, 33: 1032–1039, 1997 9383625 10.1037//0012-1649.33.6.1032

[30] Sheehan DV, Lecrubier Y, Sheehan KH, Amorim P, Janavs J, Weiller E, et al The Mini-International Interview (M.I.N.I.): The development and validation of a structured diagnostic psychiatric interview for DSM-IV and ICD-10. Journal of Clinical Psychiatry 59 (S20): 22–33, 1998. 9881538

[31] Pijnenborg GM, Van der Gaag M, Bockting CL, Van der Meer L, Aleman A. REFLEX, a social-cognitive treatment to improve insight in schizophrenia: study protocol of a multi-center RCT. BMC psychiatry 11: 161–169, 2011 10.1186/1471-244X-11-161 21975132 PMC3222612

[32] Van der Meer L, De Vos AE, Stiekema APM, Pijnenborg GHM, Van Tol M, Nolen WA, et al Insight in Schizophrenia: Involvement of Self-Reflection Networks? Schizophr Bull, 39(6), 1352–1362. 10.1093/schbul/sbs122 23104865 PMC3796073

[33] Birchwood M, Smith JD, Drury V, Healy J, Macmillan F, Slade M. A self-report insight scale for psychosis: reliability, validity and sensitivity to change. Acta Psychiatrica Scandinavica 89: 62–67, 1994 7908156 10.1111/j.1600-0447.1994.tb01487.x

[34] Sibitz I, Unger A, Woopmann A, Zidek T, Amering M. Stigma resistance in patients with schizophrenia. Schizophrenia Bulletin 37: 316–323, 2011 10.1093/schbul/sbp048 19487336 PMC3044638

[35] Ritsher JB, Otilingam PG, Grajales M. Internalized stigma of mental illness: psychometric properties of a new measure. Psychiatry Research 121: 31–49, 2003 14572622 10.1016/j.psychres.2003.08.008

[36] Kay SR, Fsizbein A, Opler LA. The Positive and Negative Syndrome Scale (PANSS) for schizophrenia. Schizophrenia Bulletin 13: 261–276, 1987 3616518 10.1093/schbul/13.2.261

[37] CBS Integrale veiligheidsmonitor 2009. Landelijke rapportage [Safety monitor 2009. National report]. The Hague, Centraal Bureau voor de Statistiek, 2010.

[38] Bureau Veiligheidsmonitor, Integrale veiligheidsmonitor (IVM) [Safety monitor]. The Hague, Nicis Institute 2010.

[39] Van Dijk JJM, van Kesteren J, Smit P. Criminal Victimisation in International Perspective, Key findings from the 2004–2005 ICVS and EU ICS. The Hague, Boom Juridische Uitgevers, 2008.

[40] Van Busschbach JT. Reports of incidents with violence in routine outcome assessments in mental health care: a prevalence study with a focus the validity of screening and clinical implications. Unpublished data, 2014.

[41] Lecomte T, Corbiere M, Laisne F. Investigating self-esteem in individuals with schizophrenia: relevance of the Self-Esteem Rating Scale-Short Form. Psychiatry research 143: 99–108, 2006 10.1016/j.psychres.2005.08.019 16725210

[42] Ullman JB. Structural Equation Modeling In: Tabachnick BG. & Fidell LS (eds). Using multivariate statistics. Boston, MA: Allyn and Bacon; 653–771, 2001.

[43] Jöreskog KG. Testing structural equation models In: Bollen KA, Long JS (eds): Testing structural Equation models. In sage Publications, New Delhi, 294–316, 1993.

[44] Tucker LR, Lewis C. A reliability coefficient for maximum likelihood factor analysis. Psychometrika 38: 1–10, 1973.

[45] Bentler PM. Comparative fit indexes in structural models. Psychological Bulletin 107: 238–246, 1990 2320703 10.1037/0033-2909.107.2.238

[46] Chapple B, Chant D, Nolan P, Cardy S, Whiteford H, McGrath J. Correlates of victimisation amongst people with psychosis. Soc Psychiatry Psychiatr Epidemiol 39: 836–840, 2004 15669665 10.1007/s00127-004-0819-4

[47] Silver E.. Mental disorder and violent victimization: The mediating effect of involvement in conflicted social relationships. Criminology, 41, 191–212, 2002.

[48] Hiday VA, Swanson JW, Swartz MS, Borum R, Wagner HR. Victimization: A link between mental illness and violence? International Journal of Law and Psychiatry, 24, 559–572, 2001 11795220 10.1016/s0160-2527(01)00091-7

[49] Silver E, Piquero AR, Jennings WG, Piquero NL, Leiber M: Assessing the violent offending and violent victimization overlap among discharged psychiatric patients. Law Hum Behav 35:49–59, 2011 10.1007/s10979-009-9206-8 20145985

[50] Ventura J, Subotnik KL, Ered A, Gretchen-Doorly D, Hellemann GS, Vaskinn A, et al: The relationship of attitudinal beliefs to negative symptoms, neurocognition, and daily functioning in recent-onset schizophrenia. Schizophrenia Bulletin 40: 1308–18, 2014 10.1093/schbul/sbu002 24561318 PMC4193707

[51] Mittal D, Sullivan G, Chekuri L, Allee E, Corrigan PW. Empirical studies of self-stigma reduction strategies: A critical review of the literature. Psychiatric Services 63: 974–981, 2012 10.1176/appi.ps.201100459 22855130

[52] Hiday VA, Swartz MS, Swanson JW, Borum RH. Wagner R. Impact of outpatient commitment on victimization of people with severe mental illness. American Journal of Psychiatry 159: 1403–1411, 2002 10.1176/appi.ajp.159.8.1403 12153835

[53] Perese EF. Stigma, Poverty and Victimization: Roadblocks to recovery for individuals with Severe Mental Ilness. Journal of the American Psychiatric Nurses Association 13: 285–295, 2007.

[54] Jonikas JA, Cook JA. Safe, secure and street-smart: Empowering women with mental illness to achieve greater independence in the community. Tresholds National research and Training Center Chicago, 1993.

[55] Corrigan PW, Holmes EP. Patient identification of “Street Skills” for a Psychosocial training Module. Hospital and Community psychiatry 45: 273–276, 1994 8188203 10.1176/ps.45.3.273

[56] Holmes EP, Corrigan PW, Stephenson J, Nugent-Hirschbeck J. Learning “Street Skills” for an Urban Setting. Psychiatric Rehabilitation Journal 20: 64–66, 1997.

[57] Lam JA, Rosenheck R. The effect of victimization on clinical outcomes of homeless persons with serious mental illness. Psychiatric Services 49: 678–683, 1998 10.1176/ps.49.5.678 9603576

