# Supplementary material for: Self-Stigma and Its Relationship with Victimization, Psychotic Symptoms and Self-Esteem among People with Schizophrenia Spectrum Disorders
Source: PLoS One. 2016 Oct 26;11(10):e0149763. doi: 10.1371/journal.pone.0149763 (PMC5082660; doi:10.1371/journal.pone.0149763)
Supplement: S1 Datafile — (RTF) [file pone.0149763.s001.rtf]

Supprting Information with

Self-stigma and its relationship with victimization, psychotic symptoms and self-esteem among people with schizophrenia spectrum disorders.


Datafile with variables 
ISMISS SERS-POS SERS-NEG PANSS POS PANSS-NEG VICTIMISATION
47	53	42	19	7	1
53	39	43	17	14	1
43	56	41	10	9	0
56	39	46	12	25	0
52	34	26	14	14	0
58	41	38	18	16	0
62	41	54	17	10	1
59	64	30	10	13	0
45	58	16	10	8	1
26	53	16	12	14	1
39	51	22	11	10	0
36	51	21	13	16	0
27	47	15	11	16	0
24	52	14	7	13	0
57	40	44	17	26	1
43	42	19	12	20	0
47	45	32	10	13	0
37	46	18	8	10	0
53	48	24	10	21	0
54	46	30	10	13	0
39	55	27	9	11	0
44	51	29	7	9	0
60	39	51	10	16	0
36	57	27	7	7	0
41	45	27	13	14	0
25	61	18	7	14	0
28	57	17	8	7	0
62	46	48	16	13	0
49	41	23	15	11	0
51	44	19	11	16	0
53	49	35	9	13	0
53	45	43	12	22	0
57	46	44	15	10	0
42	39	30	12	7	0
70	33	41	13	26	0
47	39	26	13	26	0
42	53	22	15	8	1
47	58	30	14	17	0
37	70	10	13	10	0
29	48	18	14	12	0
37	62	29	16	7	0
52	34	46	11	8	1
46	39	29	13	10	0
55	57	50	15	20	0
37	64	21	11	9	0
53	54	22	11	7	0
55	28	34	14	15	0
53	42	38	20	28	0
56	49	49	16	18	0
34	51	34	20	22	1
53	44	33	14	23	0
53	41	47	12	10	0
41	54	15	16	16	0
47	41	34	18	21	0
58	50	44	15	17	1
46	45	40	19	16	0
56	47	39	15	11	0
51	51	38	18	15	0
63	44	41	20	29	1
50	58	20	13	11	0
43	47	33	16	10	0
47	51	27	14	11	0
25	61	14	13	9	0
54	65	22	16	15	0
48	57	30	12	14	0
61	46	41	15	13	1
24	64	10	15	11	0
47	46	45	16	11	1
41	33	31	20	10	0
33	51	22	15	10	0
46	46	51	14	16	1
52	44	38	18	20	0
52	40	28	17	11	0
50	41	26	18	21	1
45	44	26	17	11	1
59	50	41	16	11	1
43	41	27	19	20	0
58	39	38	20	16	0
72	48	37	18	15	1
41	54	14	19	9	1
57	29	54	17	17	0
36	59	18	15	14	1
55	48	18	19	19	0
59	45	31	22	9	0
50	46	37	16	9	1
55	41	41	15	20	1
48	64	27	13	9	0
49	39	40	19	16	0
43	36	30	24	9	0
52	41	54	24	12	0
53	46	32	30	25	1
47	43	48	15	14	0
77	27	40	19	21	0
60	44	35	23	13	1
65	34	48	20	9	1
47	54	46	15	9	1
40	42	32	22	18	1
30	70	16	34	24	1
32	44	16	28	7	0
48	28	24	23	11	0
77	10	39	19	18	0
